# Primary cystic echinococcosis of the peritoneum: a case report

**DOI:** 10.1186/s13256-023-04289-y

**Published:** 2023-12-14

**Authors:** Nizar Kardoun, Sami Fendri, Haitham Rejab, Mejdoub Youssef, Ahmed Tlili, Meriam Triki, Nozha Toumi, Ayman Trigui, Salah Boujelbene

**Affiliations:** 1 General Surgery Department, Habib Bourguiba Hospital, University of Sfax, 0.5 Km El Ain Street, 3021 Sfax, Tunisia; 2 General Surgery Department, University Hospital of Gabes, University of Sfax, Sfax, Tunisia; 3 Pathology Department, Habib Bourguiba Hospital, University of Sfax, 0.5 Km El Ain Street, 3029 Sfax, Tunisia; 4 Department of Radiology, Habib Bourguiba Hospital, University of Sfax, 0.5 Km El Ain Street, 3029 Sfax, Tunisia

**Keywords:** Peritoneal, Cystic echinococcosis, Surgery, Case report

## Abstract

**Background:**

Peritoneal cystic echinococcosis happens usually after traumatic rupture or after surgical treatment. Primary peritoneal cystic echinococcosis is a very rare case that constitutes a diagnostic and therapeutic challenge.

**Case report:**

A 30-year-old Tunisian man was admitted for hypogastric pain since 4 months. He has a 10 cm hypogastric mass. Biological-tests were normal. A computed tomography Scan showed a cystic mass on the pelvis measuring 13 × 17 cm without echinococcosis cyst in the liver.

The patient was operated and we found a cystic mass of 17 cm located on the Douglas cul-de-sac that suggest a pelvic hydatid cyst. We have performed an aspiration of the cyst confirms the diagnosis followed by injection of hypertonic solution, extarction of the germinal layer and a maximal reduction of the pericyst. The postoperative course was uneventful.

**Conclusion:**

Trough our case, we try to focus on the diagnosis and therapeutic options of this rare entity that we should think of in front of a patient with isolated peritoneal cyst especially in endemic country.

## Introduction

The liver is the most common organ to be concerned by Echinococcus granulosus infestation but other organs can be affected at the same time or as a primary target organs. Peritoneal hydatidosis is rare (less than 13% of intraabdominal hydatidosis) but primary one are more rare (less than 2%) and should be raised in case of a cystic lesion found on computed tomography (CT) in a patient from an endemic country that represents a diagnostic and therapeutic challenge. It can be asymptomatic or can present with symptoms caused by the cyst compression effect.

## Case

A 30-year-old Tunisian man was admitted for hypogastric pain since 4 months without known medical/surgical diseases. On examination, he was afebrile and an hypogastric, 10 cm, tender and hard mass without signs of rectal or bladder compression, no more abnormality were found. White blood cells, ionogram, urea and C-Reactive protein were normal. Hydatid serology was not done.

**Fig. 1:**
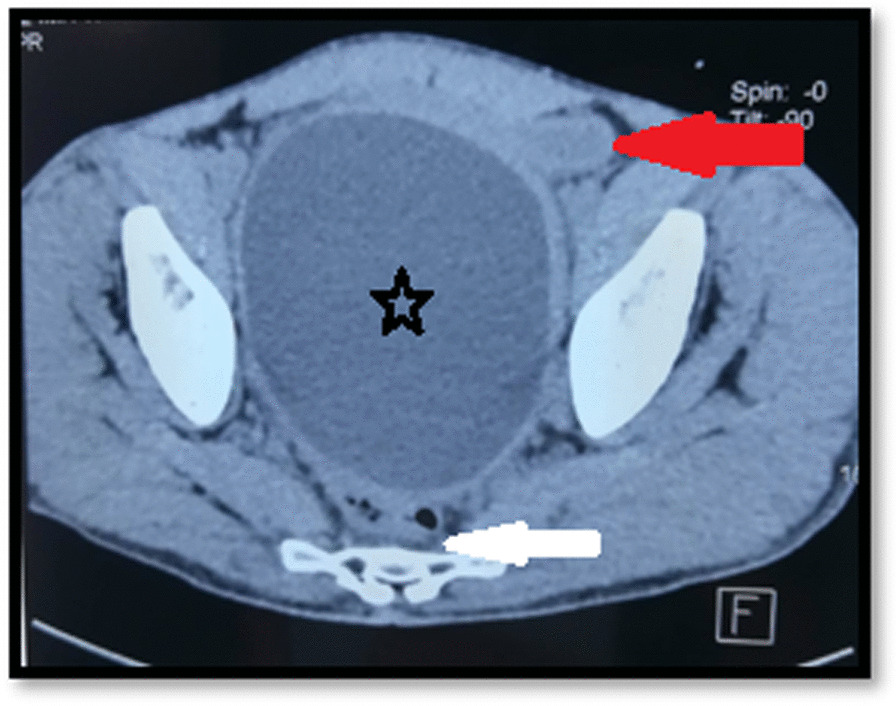
Computed tomography findings: 13 × 17 cm cyst with its own wall and homogeneous liquid content (Star) in front of the rectum (white arrow) and behind the bladder (red arrow)

A CT Scan showed a 13 × 17 cm cystic mass on the pelvis with its own wall, slightly enhanced after contrast-product injection, with homogeneous liquid content, which did not communicate with the digestive lumen and pushed the bladder forward and the colon posteriorly suggesting an ileal duplication, cystic lymphangioma or a primary peritoneal cystic echinococcosis despite the absence of daughter cysts (regarding we are an endemic country of hydatid cyst) (Fig. 1). Also, there was no echinococcosis cyst in the liver.

**Fig. 2:**
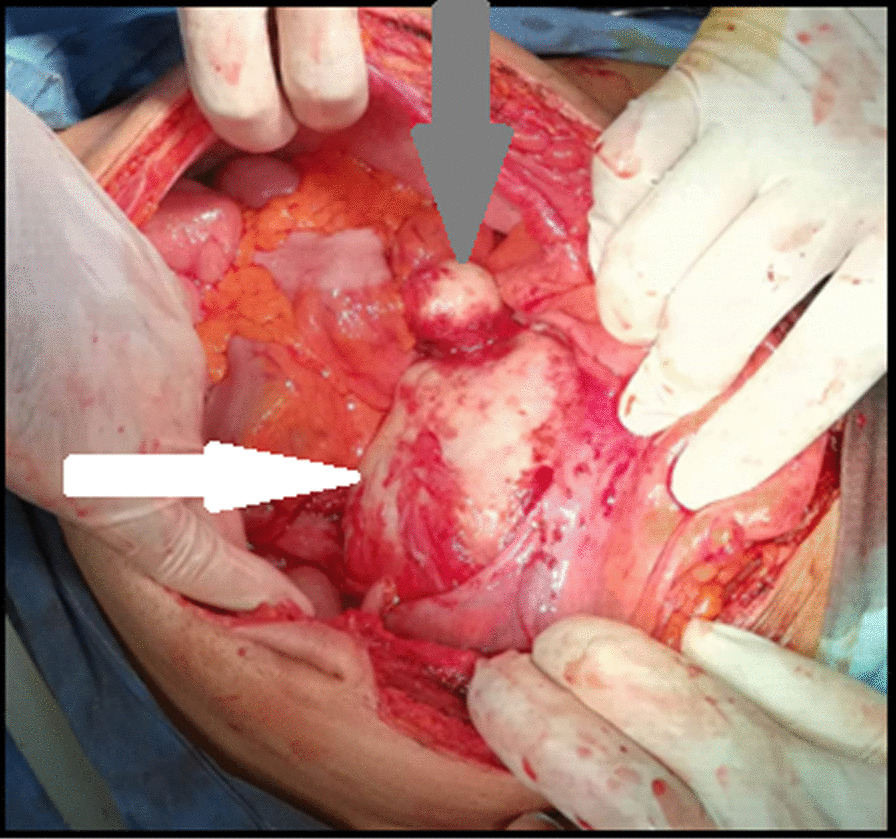
Exploration per operatively: cystic mass (white arrow) of 17 cm located on the Douglas cul-de-sac adhering to the bladder with an exovesiculation (grey arrow)

**Fig. 3:**
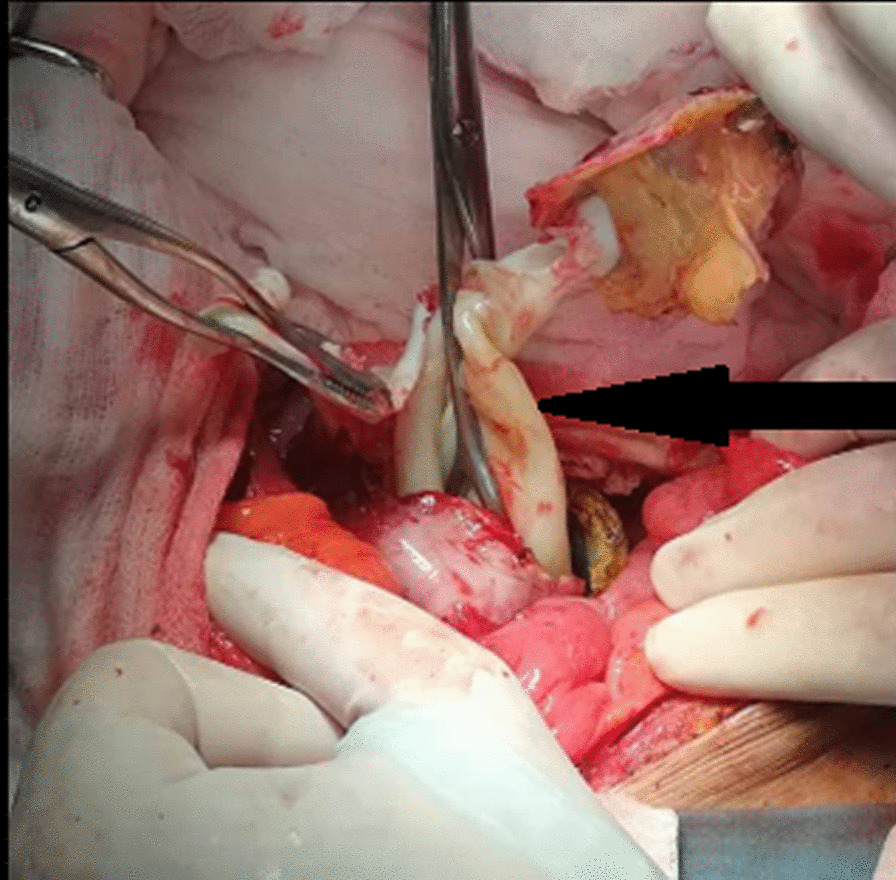
Operative view: extarction of the germinal layer (arrow)

The patient was operated-on with midline laparotomy. Intraoperative exploration found a whitish cystic mass of 17 cm located on the Douglas cul-de-sac adhering to the bladder that suggest a pelvic hydatid cyst with an exovesiculation (Fig. 2). After protecting the abdominal cavity with sponges soaked with scolicidal agents (hypertonic saline), an aspiration of the cyst confirms the diagnosis followed by injection of hypertonic solution, extraction of the germinal layer and a reduction of the pericyst by half: we left a part of pericyst witch is adhering to the bladder and the rectum (Fig. 3). Exploration of the peritoneal cavity found no other hydatid locations. The postoperative course was uneventful and patient was treated with albendazole for 6 months without recurrence. Histopathology confirms the diagnosis of cystic echinococcosis.

## Discussion

We report the rare case of a man from an endemic country of hydatidosis operated of a primary cystic echinococcosis of the peritoneum that represents a diagnostic and therapeutic challenge.

Hydatid disease is a parasitic desease caused by Echinococcus granulosus. The most common affected organs are liver (70%) and lung (13%) [1]. Primary peritoneal cystic echinococcosis is very rare [2, 3]. The dissemination is not well known but authors suggest it may occurs through lymphatic or systemic circulation [3]. Physical barriers to the diffusion of cysts are created by the liver and lungs. This could explain the reason for the low prevalence of echinococcosis of the peritoneum [4]. Peritoneal echinococcosis happens usually after spontaneous rupture or accidentally after surgical treatment [5, 6]. The diagnosis is difficult to confirm since none other hydatid cyst or daughter cysts is found and other diagnosis could be suggested such as cystic lymphangioma [7].

Clinical symptoms are not specific. Patients may suffer from signs of urinary, digestive or vascular compression. The diagnosis is based on radiological findings and serologic tests [8]. Ultrasonography may show a multilocular anechoic cystic mass that is very in favor of an hydatid disease [9]. On CT Scan, the most specific lesions are calcifications in its wall, multivesicular content and the germinal layer that could be detached from the wall. The diagnosis of our case was supported regarding the patient is from an endemic country (incidence up to 15/100.000).

Surgery is the optimal treatment; the type of intervention depends on the location of the peritoneal cyst, its size and other organs involvment. Total surgical excision is the best treatment but it can not always be performed [2]. It is imperative to protect the abdominal wall and viscera with sponges soaked with scolicidal agents to avoid the risk of spillage and allergic reactions [2, 9]. Partial excision is performed when we note that the removal of the peritoneal cyst is considered to do more harm than good because it isn’t malignant pathology. There is no consensus on the exact indications for anthelmintic therapy [4]. However, combination of peri operative albendazole-therapy is a useful treatment to prevent recurrences after surgery [5]. Puncture, aspiration, injection, and reaspiration (PAIR) technique is used on liver cytic echinoococcosis but not described for peritoneal localisation probably due to dissemination risk.

## Conclusion

Primary cystic echinococcosis of the peritoneum is a rare diagnosis that we should suspect in endemic country. Without considering its size, total surgical excision is the Gold standard treatment when there is no organ envasion (rectum, colon, ureter..) otherwise, a part of pericyst should be left.

## Data Availability

All data generated during the present study are included in the paper.

## Abbreviation

CT: Computed tomography
